# Supplementary material for: Content-rich biological network constructed by mining PubMed abstracts
Source: BMC Bioinformatics. 2004 Oct 8;5:147. doi: 10.1186/1471-2105-5-147 (PMC528731; doi:10.1186/1471-2105-5-147)
Supplement: Additional File 2 — The original results of the above study (non-essential files are deleted to keep the file size under the limit set by BMC bioinformatics). [file 1471-2105-5-147-S2.bz2 › chilibotAdditionalFile2/dip10/287ID1326789E1115/html/MAP2K1_RAF1.html]

 


 **MAP2K1** and **RAF1** 
  
Found 1002 abstracts in PubMed, retrieved 10.  
 

 What does Google say? 
 PDF only 
| .edu only 

---

**Interactive relationship** (e.g. stimulation, inhibition, etc)

**Inhibitory relationship**- These results, along with others obtained using respiratory chain inhibitors and respiration deficient cells, lead to the conclusion that PD98059, while effectively inhibiting the peroxynitrite induced Raf  [ **RAF1** ]  MEK  [ **MAP2K1** ]  signaling leading to ERK1 2 phosphorylation, promotes an enforced mitochondrial formation of ROS inducing ERK1 2 phosphorylation via a Raf 1  [ **RAF1** ]  independent MEK  [ **MAP2K1** ]  dependent mechanism.  Ref: 12860392 FEBS LettFEBS Lett,
**Neutral relationship**- We propose that FAK Src dependent, PAK1 mediated phosphorylation of MEK1  [ **MAP2K1** ]  on S298 is central to the organization and localization of active Raf  [ **RAF1** ]  MEK1  [ **MAP2K1** ]  MAPK signaling complexes,.  Ref: 12876277 J Cell BiolJ Cell Biol,

**Non-interactive relationship** (e.g. studied together, co-existance, homology, etc.)

- In conclusion, activation of the Ras Raf  [ **RAF1** ]  MEK  [ **MAP2K1** ]  MAPK pathway through Shc mediates RET PTC induced thyroid cell dedifferentiation.  Ref: 12853977 Oncogene, 2003
- From this we conclude that a high affinity chitin oligosaccharide receptor system exists which links to the Raf  [ **RAF1** ] , MEK  [ **MAP2K1** ]  and ERK pathway in zebrafish cells.  Ref: 12881410 GlycobiologyGlycobiology, 2003
- Finally, specific inhibitors of the Ras Raf  [ **RAF1** ]  MEK  [ **MAP2K1** ]  ERK pathway inhibit LSF modification in vivo.  Ref: 12858339 J Cell Biochem, 2003
- The Raf  [ **RAF1** ]  MEK  [ **MAP2K1** ]  inhibitor PD98059 enhances ERK1 2 phosphorylation mediated by peroxynitrite via enforced mitochondrial formation of reactive oxygen species.  Ref: 12860392 FEBS LettFEBS Lett, 2003
- The RAF  [ **RAF1** ]  MEK  [ **MAP2K1** ]  ERK MAPK signal transduction cascade is an important mediator of a number of cellular fates including growth, proliferation and survival.  Ref: 12879021 Oncogene, 2003
- These events were accompanied by down regulation of the Raf 1  [ **RAF1** ]  MEK  [ **MAP2K1** ]  ERK pathway as well as diminished expression of Bcr Abl and cyclin D1, cleavage of p21 CIP1 and pRb, and induction of the stress related kinases JNK and p38 MAPK.  Ref: 12893773 BloodBlood, 2003
- Co treating PC 3 cells with quercetin significantly attenuated EGF and TGF alpha induced growth and phosphorylation of ErbB 2, ErbB 3, c Raf  [ **RAF1** ] , MAPK kinase 1 2 MEK1  [ **MAP2K1** ]  2, MAPK, Elk 1 and Akt 1.  Ref: 12888923 Int J Oncol, 2003
- Taken together, our results identify a novel mechanism whereby a Gi o protein coupled receptor inhibits cell proliferation by stimulating ERK signaling via a SHP 1 SHP 2 PI3K Ras Rap1 B Raf  [ **RAF1** ]  MEK  [ **MAP2K1** ]  pathway.  Ref: 12878607 J Biol ChemJ Biol Chem, 2003
- Gastrin activated transcription through a Raf  [ **RAF1** ] , Mek  [ **MAP2K1** ]  and Erk dependent but Ras independent pathway.  Ref: 12891554 Gastroenterology, 2003
